# Emerging functions of pseudoenzymes

**DOI:** 10.1042/BCJ20220373

**Published:** 2023-05-19

**Authors:** Timea Goldberg, Anju Sreelatha

**Affiliations:** 1 Department of Physiology, University of Texas Southwestern Medical Center, Dallas, TX, U.S.A.; 2 Center for Mineral Metabolism and Clinical Research, University of Texas Southwestern Medical Center, Dallas, TX, U.S.A.

**Keywords:** allosteric regulation, catalytic motif, molecular scaffolds, molecular switch, protein moonlighting, substrate trap

## Abstract

As sequence and structural databases grow along with powerful analysis tools, the prevalence and diversity of pseudoenzymes have become increasingly evident. Pseudoenzymes are present across the tree of life in a large number of enzyme families. Pseudoenzymes are defined as proteins that lack conserved catalytic motifs based on sequence analysis. However, some pseudoenzymes may have migrated amino acids necessary for catalysis, allowing them to catalyze enzymatic reactions. Furthermore, pseudoenzymes retain several non-enzymatic functions such as allosteric regulation, signal integration, scaffolding, and competitive inhibition. In this review, we provide examples of each mode of action using the pseudokinase, pseudophosphatase, and pseudo ADP-ribosyltransferase families. We highlight the methodologies that facilitate the biochemical and functional characterization of pseudoenzymes to encourage further investigation in this burgeoning field.

## Introduction

Pseudoenzymes are proteins that lack conserved catalytic amino acids when compared with their catalytically active counterparts. Once considered inactive or dead, these ‘zombie enzymes' carry out a large variety of non-enzymatic functions across the tree of life, in addition to occasional enzymatic functions [1]. The abundance of pseudoenzymes has become apparent as increasingly powerful analytical tools examine growing sequence and structural databases. While the precise number of pseudoenzymes has yet to be determined, especially given the potential for misidentification, pseudoenzymes are present in over 20 enzyme families (Table 1) [1–4]. Phylogenetic mapping suggests that pseudoenzymes may have evolved from enzymes through a loss of catalytic residues and/or structural rearrangements that hinder substrate binding [4,5]. Although less common, there are also examples of enzymes that evolved from pseudoenzymes through the gain of catalytic residues [4]. Here, we will review several pseudoenzyme families, with emphasis on the pseudokinases, pseudophosphatases, and pseudo ADP-ribosyltransferases (pseudoARTs), in addition to the technological advances that have facilitated the study of a multitude of functions.

**Table 1.** Methods for functional analysis of pseudoenzymes

Mode of action	Family	Examples	Methods of study	References
Allosteric regulation	Pseudokinase	Pseudokinase FAM20A increases the activity of the secretory pathway kinase FAM20C via heterodimerization	X-ray crystallography, thermal stability shift assay, co-immunoprecipitation, size exclusion chromatography, *in vitro* kinase activity assay, mutational analysis, cellular activity assay	[44,47]
Allosteric regulation	Pseudophosphatase	Pseudophosphatase MTMR13 binding increases the activity of the lipid phosphatase MTMR2	co-immunoprecipitation, negative stain electron microscopy, size exclusion chromatography, *in vitro* phosphatase activity, subcellular co-localization	[117]
Allosteric regulation	Pseudoprotease	Pseudoprotease cFLIP binds procaspase-8 to regulate apoptosis	co-immunoprecipitation, *in vitro* protease activity, mutational analysis, cellular activity assay	[118,119]
Allosteric regulation	Pseudodeubiquitinase	Pseudodeubiquitinase Rpn8 promotes the deubiquitinase activity of Rpn11	Cryo-electron microscopy, X-ray crystallography, mutational analysis, *in vitro* isopeptidase activity assay	[120,121]
Allosteric regulation	Pseudoligase (E2)	The pseudodomain of Mms2 binds E2 ligase Ubc13 to position ubiquitin and promote activity	X-ray crystallography, mutational analysis, competition assay, *in vivo* diubiquitin synthesis assay	[122]
Allosteric regulation	Pseudonuclease	Pseudoendoribonuclease CPSF-100 complexes with active CPSF-73 to function in the pre-mRNA 3′ end processing complex	X-ray crystallography, *in vitro* endonuclease activity assay	[123,124]
Allosteric regulation	PseudoGTPase	PseudoGTPases Rnd1 and Rnd3 bind and activate the GAP activity of p190RhoGAP	yeast two-hybrid assay, *in vitro* pulldown assay, *in vitro* GAP assay, mutational analysis, co-immunoprecipitation, fluorescence microscopy of actin morphology	[125]
Allosteric regulation	Pseudolyase	The inactive homolog S-adenosylmethionine decarboxylase potently activates S-adenosylmethionine decarboxylase through heterodimerization	Steady-state kinetic analysis, mutational analysis, sedimentation equilibrium analytical centrifugation, X-ray crystallography, kinetic analysis of inhibitor binding	[126,127]
Allosteric regulation	Pseudophospholipase	Phospholipase A2 inhibitor is a pseudophospholipase from snake venom that inhibits the human A2 phospholipase via head-to-tail heterodimerization	X-ray crystallography	[128]
Allosteric regulation	Pseudooxidoreductase	Inactive variant of ALDH2 inhibits the activity of wild-type ALDH2 by destabilizing the Heterotetramer	X-ray crystallography, gel filtration chromatography, *in vitro* oxidoreductase activity assay, mutation analysis	[129,130]
Allosteric regulation	Pseudo arginine methyltransferase	TbPMRT1 PRO stabilizes and promotes the methyltransferase activity of TbPRMT1 ENZ by forming a heterotetramer	SEC-SAXS, X-ray crystallography, mutational analysis, co-immunoprecipitation, immunofluorescence microscopy	[109,110]
Molecular switch	Pseudokinase	Phosphorylation of the pseudokinase MLKL results in a conformation change that releases the N terminal auto-inhibition, promotes oligomerization and translocation to the membrane	Domain truncation analysis, X-ray crystallography, thermal stability shift assay, native mass spectrometry, phenotypic characterization of knockout cell lines and mice, mutational analysis, liposome dye release assay, cell death assay, protein interaction assays, SAXs, surface plasmon resonance	[62,65]
Signaling scaffold	Pseudokinase	The pseudokinase TRIB1 recruits the E3 ligase, COP1, and its substrates to facilitate ubiquitination	X-ray crystallography, *in vitro* pulldown, isothermal titration calorimetry, mutational analysis, thermal shift assay, SAXS, SEC-MALLS, fluorescence polarization displacement, enzyme-linked immunosorbent assay	[70,72]
Signaling scaffold	Pseudodeubiquitinase	Spliceosomal protein PRP8 functions as a scaffold for spliceosome formation.	X-ray crystallography, yeast two-hybrid screen, mutational analysis, CD spectroscopy, *in vitro* pulldown	[131,132]
Signaling scaffold	Pseudoligase (E2)	Ubiquitin E2 variant domains in BRCC45 promote protein interaction in BRISC-SHMT2 and BRCA1-A	size exclusion chromatography, *in vitro* deubiquitylation assay, mutational analysis, negative stain electron microscopy, cryo-electron microscopy, co-immunoprecipitation, thermal shift assays, native mass spectrometry, analytical size exclusion chromatography	[133]
Signaling scaffold	PseudoGTPase	PseudoGTPase CENP-M is important for assembly and stability of kinetochore assembly	co-immunoprecipitation, analytical size exclusion chromatography, nucleotide binding assay, X-ray crystallography, negative stain electron microscopy, mutational analysis, *in vitro* pulldown, cellular phenotype characterization in siRNA depleted	[134]
Signaling scaffold	Pseudotransferase	Pseudotransferase MiD51 acts as a scaffold for the recruitment of Drp1 for mitochondrial fission	cellular localization using immunofluorescence, X-ray crystallography, isothermal titration calorimetry, mitochondrial morphology analysis using immunofluorescence, mutational analysis, immunoprecipitation	[135]
Competitive inhibition	Pseudophosphatase	Pseudophosphatase STYX competes with DUSP4 phosphatase by binding the same substrate, ERK2	mutational analysis, in cell MAPK activation assays, fluorescence recovery after photobleaching, co-immunoprecipitation, *in vitro* pulldown assay	[82]
Competitive inhibition	Pseudodismutase	SOD homologs from *Shope fibroma* virus bind copper chaperones to block SOD1 activation	mutational analysis, *in vitro* superoxide dismutase metal binding analysis, *in vitro* pulldown, co-immunoprecipitation, cellular localization using immunofluorescence	[136]

Examples of each mode of action in diverse families of pseudoenzymes with the goal of highlighting a few common methods used for pseudoenzyme studies. The information presented is limited in range with additional examples reviewed in [1,5]. ALDH2, aldehyde dehydrogenase 2; BRCA-1, breast cancer gene 1; BRCC, brain and reproductive organ expressed; BRISC, BRCC36 isopeptidase complex; CENP, centromere protein; cFLIP, cellular FLICE-like inhibitory protein; CPSF, cleavage and polyadenylation specificity factor; COP1, constitutive photomorphogenesis protein 1; DUSP, dual-specificity phosphatase; Drp1, dynamin-related protein; ERK, extracellular signal-regulated kinase; FAM, family with sequence similarity; GAP, GTPase activating protein; PRP8, pre-mRNA processing; TbPMRT1, *Trypanosome brucei* protein arginine methyltransferase; MiD51, mitochondrial dynamics proteins of 51 kDa; MLKL, mixed lineage kinase domain-like; MTMR, myotubularin-related protein; SHMT2, serine hydroxymethyltransferase 2; SOD1, superoxide dismutase; STYX, serine/threonine/tyrosine interacting protein.

## Pseudokinases

Identification of pseudoenzymes relies on computational methods to detect proteins that diverge at highly conserved catalytic residues based on sequence or structural alignments. A major limitation of such computational methods is the fact that they rely on our current understanding of the catalytic mechanisms. One of the most well-studied signaling families is the kinase superfamily, consisting of protein and small molecule kinases. In 1988, Hanks et al. [6] generated a sequence alignment of 65 protein kinases to reveal conserved domains present in protein kinases. The essential domains are the VAIK motif in which the lysine orients the ATP in the active site, the HRD motif in which the aspartate acts as a nucleophilic base that catalyzes phosphotransfer, and the DFG motif in which the aspartate binds the metal ion to co-ordinate the ATP in the active site. A broad sequence analysis throughout the domains of life revealed that ∼98% of eukaryotic, 6% of bacterial, and 2% of archaeal proteomes harbor pseudokinases as defined by the absence of one or more of the three conserved domains [7].

The kinome comprises of all the protein kinases identified in a genome [8]. In eukaryotes, the proportion of pseudokinases present in kinomes vary between vertebrate and non-vertebrate species. Consistent with the vertebrate kinomes, ∼10% of the human kinases are pseudokinases [8]. In contrast, more than half of the kinomes of protozoan parasites, *Plasmodium falciparum* and *Giardia lamblia* consist of pseudokinases [7]. Bacterial and archaeal proteomes display more variance in the size of kinomes and pseudokinomes than eukaryotic proteomes. While some bacterial and many archaeal proteomes have no detectable kinases or pseudokinases, seven out of eight kinases in *Halorientalis regularis* are pseudokinases, highlighting the diversity in the size of pseudokinomes [7].

The kinome is divided into groups based on sequence, structural, functional, and evolutionary similarity [8,9]. The prevalence of pseudokinases varies across the kinome with large expansions in certain groups of kinases. The tyrosine kinase-like (TKL) group harbors the largest number of pseudokinases that are predominant in plants and fungi [7–10]. The biological role and functional importance of the pseudokinase expansion, particularly in the family of interleukin-1 receptor-associated kinases (IRAK) of TKL group, is unclear. The domain architecture of some archael, bacterial, and eukaryotic pseudokinases contain accessory domains, such as leucine-rich repeat domains, that may play a role in ligand binding, and hence provide insights into their putative function [7,11]. The human pseudokinases are present in all groups of kinases; approximately half of the human pseudokinases have orthologs in flies, worms, and mice [5,7,8,12,13]. These model organisms provide a platform for genetic and functional studies as pseudokinases emerge as significant players in human pathophysiology [12,14,15].

One example of a pseudokinase with ties to pathophysiology is with no lysine (WNK1) kinase, which lacks the conserved lysine in the VAIK motif. Mutations in WNK1 are associated with autosomal dominant pseudohypoaldosteronism characterized by hyperkalemia and hypertension [16]. WNK1 was initially isolated in an attempt to identify members of the Mitogen-activated protein kinase kinase (MEK) family in rat cDNA libraries [17]. To better understand the molecular mechanisms of diseases caused by WNK1 mutations, interacting proteins were immunoprecipitated with endogenous WNK1 from testis extract. WNK1 binds and phosphorylates STE20/SPS1-related proline/alanine-rich kinase (SPAK) and oxidative stress response kinase-1 (OSR1) *in vitro* [18]. Phosphorylation leads to the activation of OSR1 and SPAK which then phosphorylates and stimulates the activity of Na^+^K^+^2Cl^−^ cotransporter (NKCC1) [18,19]. The discovery of WNK1 phosphorylation activity suggests that it is an atypical kinase rather than a pseudokinase. Structural analysis revealed the migration of the conserved lysine from the β3 VAIK motif to the β2 strand. Mutation of the catalytic β2 lysine abolishes WNK1 activity, suggesting non-canonical migration of the essential lysine leading to an atypical mechanism of catalysis [20].

Similarly, the protein-O mannose kinase (POMK), also known as SGK196, is an atypical kinase once predicted to be catalytically inactive [8]. POMK lacks all three of the conserved catalytic motifs including the aspartates in both the HRD and DFG, the lysine in the VAIK motif, as well as the conserved glutamate that forms a salt bridge with the lysine [8,21]. Despite these missing residues, POMK catalyzes the phosphorylation of the C6 hydroxyl position in O-mannose linked to α-dystroglycan [21]. Mutations in POMK that inhibit phosphorylation of the GalNAc-β3-GlcNAc-β4-Man linked to α-dystroglycan alter the ability of α-dystroglycan to bind to the extracellular matrix protein, laminin, leading to congenital and limb-girdle muscular dystrophies [21]. As with WNK1, the missing lysine in the VAIK is present in the β2 strand; mutation of the migrated lysine abolishes kinase activity in both human and zebrafish POMK [22]. Notably, introducing the lysine back into the VAIK motif along with the mutation of the β2 lysine to glycine improves the catalytic activity in comparison with wild-type POMK [22]. Structural and biochemical analysis of *Danio rerio* POMK illustrates that the migrated DLD and MCD motifs serve the roles of the canonical DFG and HRD motifs, respectively [22]. Consistently, expression of wild-type POMK, but not the catalytic mutants, in POMK knockout cells, rescues binding of α-dystroglycan to laminin. While cases of such catalytic activity in pseudoenzymes are not common, these studies demonstrate the importance of biochemical, structural, and functional analysis of sequence-based predictions of inactivity with the added incentive of potentially identifying new mechanisms of regulation and catalysis. A few pseudokinases, including Janus kinase (JAK) pseudokinase domain JH2 and CASK, demonstrate low or impaired kinase activity, although the functional importance of catalytic activity in cells is controversial [23–26].

Excitingly, recent studies have demonstrated that certain pseudokinases may catalyze alternative transferase activities. The pseudokinase SelO is conserved from bacteria to humans, suggesting an important role in biology [27,28]. This evolutionary conservation provides multiple advantages in the structural and biochemical analysis of this unique pseudokinase. The structure of the *Pseudomonas syringae* SelO revealed that ATP binds in an inverted orientation in the active site when compared with canonical kinases [29]. Structure-guided biochemical analysis revealed that SelO catalyzes the transfer of AMP to protein substrates in a post-translational modification known as AMPylation. This modification was validated using radiolabeled ATP, immunoblotting with AMP-protein specific antibody, and mass spectrometry analysis [29]. Given the orientation of ATP in the active site, an ATP analog labeled with a biotin at the N6 position of adenine facilitated the enrichment of AMPylated substrates. SelO AMPylates multiple mitochondrial proteins, particularly proteins involved in redox homeostasis. Congruently, yeast lacking SelO have reduced viability in response to oxidative stress. Collectively, these studies revealed the first instance of an alternative transferase activity catalyzed by the kinase fold. Following this finding, SidJ, a bacterial effector pseudokinase was shown to catalyze protein polyglutamylation [30–34]. Notably, the NIRAN domain of coronavirus protein nsp12, which harbors sequence similarity to SelO, catalyzes RNAylation [35–37]. These alternative transferase activities have been extensively reviewed in [38]. As demonstrated in the above studies, intact mass analysis of the substrates is a powerful technique for identifying unexpected and novel post-translational modifications catalyzed by pseudoenzymes. Intact mass analysis can reveal the mass increment or decrement as well as diverse post-translational modifications present on a protein [39]. In line with the top-down proteomics approach, MS/MS fragmentation can then be used to identify the site and relative abundance of the modification [40].

Pseudokinases with enzymatic activity could arguably be termed atypical kinases, although many have significantly decreased enzymatic activity in comparison with canonical kinases with conserved architecture [23,24]. Based on these observations, Zeqiraj and van Aalten [41] classified predicted pseudokinases into three enzymatic groups: inactive pseudokinases, low-activity kinases or active pseudokinases. The majority of pseudokinases perform non-catalytic functions, even among those that have some *in vitro* activity [42]. One such non-catalytic function of pseudoenzymes is the regulation of active enzyme counterparts [3]. An example is the pseudokinase Fam20A, which belongs to the family of secretory pathway kinases including Fam20B and Fam20C [43]. Fam20A lacks a conserved catalytic glutamate present in the Fam20C family [44]. Mutations in Fam20A, as well as Fam20C, result in defects in biomineralization [45]. However, Fam20C was shown to phosphorylate 75% of the secreted phosphoproteome while Fam20A lacks kinase activity [44,46]. *In vitro* and in cell co-expression studies demonstrate that the expression of Fam20A increases the activity of Fam20C. Structural analysis revealed the mechanisms by which Fam20A increases the catalytic efficiency of Fam20C through the formation of Fam20A–Fam20C heterodimer [44,47]. Fam20A mutations in the dimer interface impair its interaction with Fam20C and subsequently do not enhance Fam20C activity [47]. Hence, the pseudokinase Fam20A acts as an allosteric activator of Fam20C through heterodimerization.

In contrast with the Fam20A and Fam20C dimer, the JAK pseudokinase domain acts as an allosteric inhibitor of JAK activity. The receptor-bound JAKs contain both a kinase (JH1) and a pseudokinase (JH2) domain in the same polypeptide as a result of domain duplication [48]. Removal of the pseudokinase domain in JAK2 and JAK3 increase their catalytic activity [49]. Congruently, many of the cancer-associated mutations of JAKs that result in constitutive activation cluster at dimer interface between the active and inactive domains [50]. However, the mechanism of JH1 inhibition by the JH2 pseudokinase domain is poorly understood, with differing evidence supporting *in cis* or *in trans* models of regulation [51]. The crystal structure of the kinase and pseudokinase domains of the JAK family member, TYK2, reveals the interaction between the N lobes of the pseudokinase and kinase domains *in cis* [50]. In contrast, small-angle X-ray scattering (SAXs) analysis of the human JAK2 support *in trans* inhibition of the JH1 kinase domain of one protein molecule by the JH2 pseudokinase domain of another protein molecule [52]. The first structure of the full-length mouse JAK1, solved in 2022 using cryo-EM microscopy, provides further insight into these two mechanisms of regulation [53]. Glassman et al. [53] utilized the constitutively active JAK1 V657F along with a nanobody to stabilize the complex of JAK1 with a truncated cytokine receptor, interferon λ receptor 1 (IFNλR1). In the resulting JAK1-IFNλR1 dimer, the pseudokinase domain interfaces *in cis* with the kinase domain and *in trans* with another pseudokinase domain [53]. Oncogenic mutations that activate JAK1 map to both interfaces. Mutations at the pseudokinase:pseudokinase interface stabilize the dimeric kinase active state while mutations at the pseudokinase:kinase interface destabilize the auto-inhibited state [53]. These results suggest that the pseudokinase domain may not only regulate JAK activity *in cis* with the kinase domain, but also via dimer formation *in trans* with the opposing pseudokinase domain [53,54]. More recently, AlphaFold was used to model the homo- and heterodimers of JAK1, JAK2, JAK3, and TYK2 and examine the amino acids at the dimeric interface that contribute to the allosteric inhibition of JAKs [54]. Collectively, these studies demonstrate how the non-catalytic functions of JAK pseudokinase domains regulate the kinase activity of JAKs. This mode of allosteric regulation is found in several other pseudokinases:kinase pairs such as HER3:EGFR, IRAK3:IRAK4, and STRAD:LKB1 [55–57]. Interestingly, the pseudokinase vaccinia-related kinase 3 (VRK3) activates the vaccinia H1-related (VHR) phosphatase, thus providing an example of allosteric regulation across enzyme families [58].

In addition to serving as allosteric regulators, pseudokinases may also act as molecular switches in signaling pathways [42]. The structure of mouse mixed lineage kinase domain-like (MLKL) revealed a four-helix bundle tethered to a pseudokinase kinase domain that lacks the catalytic and metal binding aspartate in the HRD and DFG motif, respectively [8,59]. Although mice deficient of MLKL show no overt phenotype, MLKL is necessary for necroptotic signaling [59]. Domain truncation analysis of mouse MLKL revealed that the four-helix bundle is sufficient for MLKL-mediated cell death and the pseudokinase domain is required to suppress MLKL-mediated cell death [60]. Phosphorylation of the pseudokinase domain mediated by receptor-interacting serine–threonine kinase (RIPK3) induces a conformation change that releases the four-helix bundle restrained by the pseudokinase domain [60]. Upon release, the cytoplasmic four-helix bundle inserts into the membrane to induce pore formation and necroptotic cell death [59–61]. In this scenario, phosphorylation of the pseudokinase domain of MLKL acts as a molecular switch to allow the membrane translocation of the N terminal four-helix bundle and subsequent necroptotic cell death [59–63].

Structural analysis of the pseudokinase domains of rat, mouse, horse, and human orthologues of MLKL depict conformational variations that contribute to the different mechanisms of MLKL activation in mice and humans [64]. In particular, the mechanism of activation of the human homolog of MLKL requires the stable interaction of MLKL with the necrosomal RIPK3 prior to phosphorylation, after which MLKL dissociates and moves to the plasma membrane to compromise membrane integrity [61–66]. Analysis of human MLKL conformations using synthetic binding proteins, or monobodies, demonstrate that MLKL exists in complex with RIPK3 under basal conditions. Upon induction of necroptosis, RIPK3 phosphorylates MLKL which leads to the dissociation of RIPK3–MLKL complex [62,63]. Akin to mouse MLKL, the phosphorylation of human MLKL drives a conformation change that leads to the progression of necroptosis, likely by causing dissociation from RIPK3 in addition to oligomer formation and translocation into the membrane.

A common theme in several proteins harboring the pseudokinase domain is the presence of additional accessory domains, as seen with MLKL. Another pseudokinase family that harbors accessory domains is the family of Tribbles pseudokinases (TRIB1, TRIB2, TRIB3, SgK495), which lacks the aspartate in the DFG motif and harbors an E3 ligase binding motif in the C terminus [67,68]. The E3 ligase binding motif recruits the E3 ubiquitin ligase, COP1, while the pseudokinase domain of TRIB1 binds and positions the substrates, such as the tumor suppressor CCAAT enhancer-binding protein (C/EBPα) for ubiquitination [68–70]. In the auto-inhibited state, the distorted αC helix of the pseudokinase domain binds with the E3 ligase binding domain [70,71]. C/EBPα substrate binding in the pseudokinase domain of TRIB1 induces a conformation change to release the auto-inhibited COP1 binding motif [72]. Through this mechanism, TRIB1 functions as a scaffold that brings together the COP1 ligase with substrates to facilitate ubiquitination. The related TRIB2 pseudokinase also appears to act in a similar manner, as revealed by the crystal structure of the active conformation of TRIB2 complexed with a nanobody [73]. Notably, TRIB2 is implicated in various diseases, including acute myeloid leukemia, highlighting the need for drug development [74]. Foulkes et al. [75] screened small molecules from the Published Kinase Inhibitor Set that stabilize or destabilize TRIB2 using the thermal shift assay. TRIB2 is capable of weakly binding ATP and this binding site is targeted by covalent inhibitors of EGFR kinases [75,76]. Binding of the inhibitor destabilizes TRIB2, resulting in proteosome-dependent degradation of TRIB2 [75]. In addition to identifying a potential tool to degrade TRIB2, the screen also revealed several compounds that will be beneficial in studying the cellular signaling of TRIB2 [75]. The Tribbles protein family has multiple functions in addition to acting as adaptor proteins. For example, TRIB1 may regulate the nucleocytoplasmic shuttling of COP1 via recruitment of the C terminal E3 ligase binding domain, thus adding to the complexity [77].

**Figure 1.**
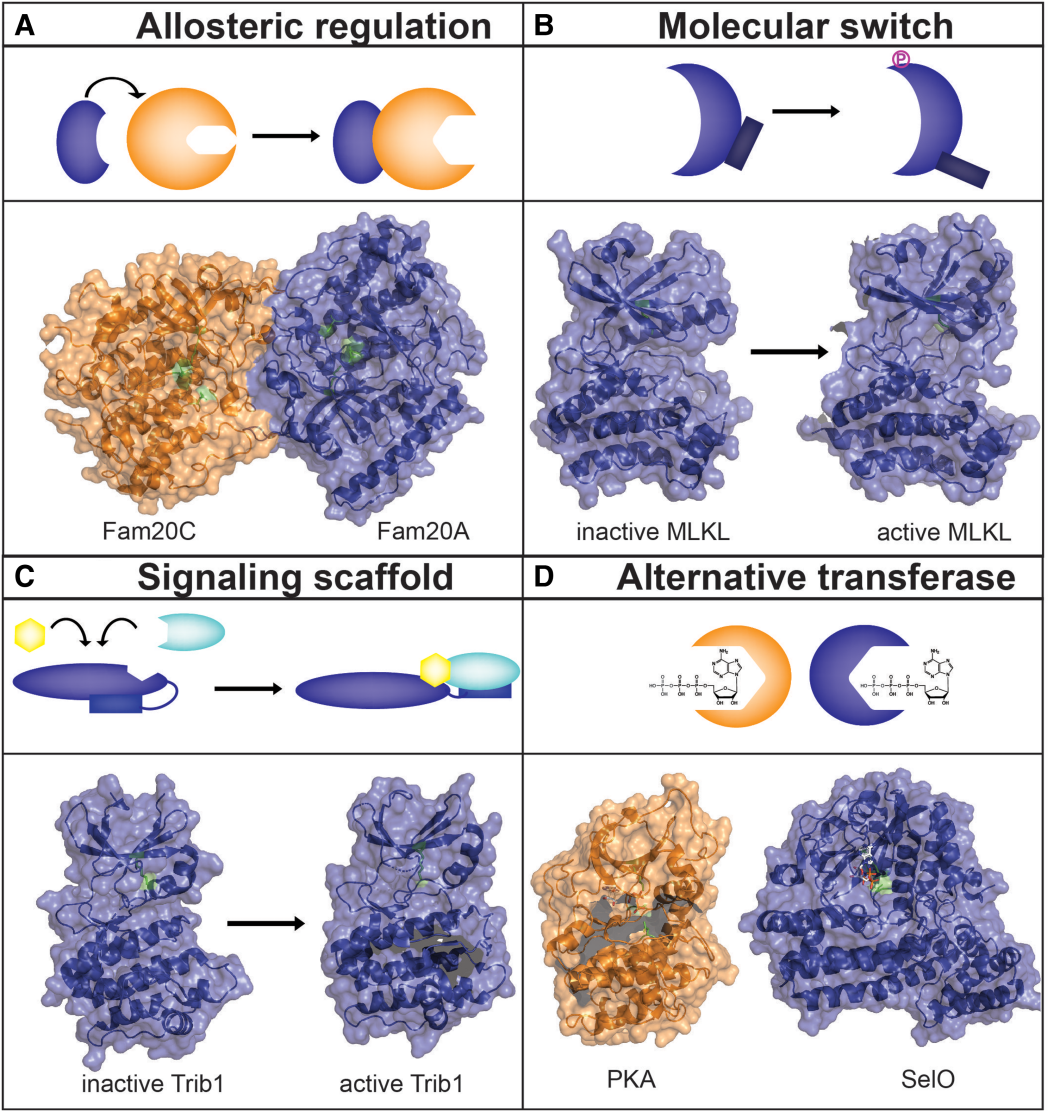
Schematic diagram depicting various modes of action of pseudokinases. (**A**) Fam20A forms a reverse face-to-face dimer with Fam20C and allosterically activates the kinase activity of Fam20C. PDB 5YH3 [115]. (**B**) MLKL acts as a molecular switch which undergoes conformational change upon phosphorylation to release the 4HB executioner domain. Although the structure of full-length human MLKL is not available to depict the movement of the four-helix bundle domain, obvious conformational changes in the kinase domain are observed in the active and inactive states. PDB 4MWI (Human inactive MLKL) [108]; PDB 7JXU (Human active MLKL) [62]. (**C**) Tribbles pseudokinase is an example of a scaffolding protein that serves as a signaling hub to facilitate ubiquitination of substates through the recruitment of substrate, depicted by yellow polygon, and E3 ligase, depicted by green sphere. PDB 5CEM (inactive Trib1) [70]; PDB 6DC0 (active Trib1) [72]. (**D**) Only one human pseudokinase has been shown to catalyze alternative transferase activity. SelO binds ATP in an inverted orientation in comparison with canonical kinases to catalyze the transfer of AMP to protein substrates in a post-translational modification known as AMPylation. PDB 1ATP (protein kinase A) [116]; PDB 6EAC (SelO) [29]. In all panels, blue depicts the pseudokinases and orange depicts the kinases. Conserved residues such as the lysine in VAIK, aspartates in HRD and DFG are shown in green. Additional examples of pseudokinases are reviewed in [42].

These studies exemplify the biochemical and functional versatility of the pseudokinase superfamily (Figure 1). Additional examples of pseudokinases that act as allosteric regulators, scaffolding proteins, and molecular switches are reviewed in [12,42].

## Pseudophosphatases

Chen et al. [78] identified 264 human phosphatases with large diversity in folds and residues that make up the catalytic motifs. Sequence-based conservation analysis was used to define 26 human pseudophosphatase domains that contain variations in one of the catalytic motifs [78]. These pseudophosphatases are predicted to lack catalytic activity, but share common mechanisms of action with pseudokinases such as competitive inhibition, allosteric regulation, and scaffolding [79]. The majority of the human phosphatases and pseudophosphatases have the Cys-based Class 1 (CC1) fold with the HCX_5_R catalytic motif [78].

Mutation of a single amino acid can revert some CC1 pseudophosphatases to active enzymes [42,80,81]. One such example is the serine/threonine/tyrosine interacting protein (STYX) which harbors the CC1 fold and belongs to the dual-specificity phosphatases (DUSPs) family [81]. STYX was initially identified in mice testis with amino acid sequence similarity to the DUSPs and was characterized as an inactive phosphatase given the lack of the conserved cysteine in the HCX_5_R motif [81]. Interestingly, restoration of the catalytic motif through the mutation of glycine 120 to cysteine revived the dephosphorylation activity [81]. The functional importance of STYX was tested using computational modeling and biochemical analysis of extracellular regulated kinase (ERK), given that other members of DUSPs are implicated in ERK inactivation [82]. Binding of ERK2 was examined by fusing complementary halves of yellow fluorescent protein (YFP) to STYX and ERK2, where the interaction of ERK2 with STYX would bring the sections of YFP together to produce a fluorescent signal. The split YFP assay along with *in vitro* pulldown assays demonstrate that STYX interacts with ERK2, predominantly in the nucleus. The ability to bind the same ligand as an active counterpart can serve multiple purposes, with such pseudoenzymes potentially restraining ligands in specific locations or serving as competitive inhibitors [79]. STYX sequesters ERK2 in the nucleus and competes with the active phosphatase, DUSP4. Hence, the pseudophosphatase STYX regulates ERK2 localization and signaling to modulate cell migration [82].

As demonstrated with STYX and other pseudophosphatases, the substrate-trapping mechanism of pseudophosphatases can be harnessed to identify binding partners [83,84]. Similarly, one could make a trapping mutation in an active enzyme to identify substrates [85]. Methods to further study the biological function of pseudophosphatases are detailed in [83,86]. An arrangement of active and pseudophosphatase domains is found in some receptor-linked protein tyrosine phosphatases, PTPR. The distal pseudophosphatase domain is important for substrate specificity, stability, and activity of the catalytically active membrane proximal phosphatase domain [87,88]. Although less studied than the pseudokinase family, the pseudophosphatase family holds the potential to reveal exciting biology given the evolutionary diversity in catalytic folds [89].

## PseudoARTs

Another family of emerging pseudoenzymes is the ADP-ribosyltransferase (ART) like proteins lacking the classical active site. ARTs catalyze the transfer of ADP ribose from NAD^+^ to substrates to form a mono-ADP ribose or poly-ADP ribose modification [90]. Members of the ART family display high similarity in structural folds despite low sequence conservation [91]. Amino acid residues that are spatially separated in primary sequence form the active site in the tertiary structure consisting of H-H-hydrophobic residue, H-Y-[QED], or R-[ST]-E motifs [91]. Using structural analysis and phylogenetic mapping, Wyzewski et al. [92] identified LRRC9-ART as a novel ART-like family predicted to be pseudoenzymes based on the lack of the catalytic motifs.

The transgene activation suppressor protein (TASOR) was recently identified to harbor a pseudoART domain that forms the human silencing hub (HUSH) complex along with M-phase phosphoprotein 8 (MPP8) and Periphilin [93]. The HUSH complex regulates trimethylation of Histone H3 lysine 9 (H3k9me3) to repress transcription. Using truncation analysis of reconstituted TASOR in cells, Douse et al. [94] mapped the function of the multidomain TASOR in the HUSH complex. TASOR acts as a scaffolding protein necessary to bring together MPP8 and Periphilin. However, the interaction was independent of the pseudoART domain. NMR structure analysis of the pseudoART domain reveals the overall ART fold with a degenerate active site, similar to the inactive PARP13. Congruently, the pseudoART domain does not bind a NAD^+^ mimetic nor catalyze poly-ADP-ribosylation *in vitro* [94]. However, the pseudoART domain is necessary for transgene repression through an unknown mechanism.

The ART family provides clues about the evolutionary origin of pseudoenzymes, suggesting that pseudoenzymes may have evolved from catalytically active ancestors. Shannon's entropy analysis of ARTs in the secreted proteins of a fungal phytopathogen, *Magnaporthe oryzae*, provided a numerical estimate of the degree of divergence from an estimated consensus sequence for each protein and mapped the most conserved residues [95]. ARTs predicted to be pseudoenzymes diverge from the consensus more so than active ARTs but tend to share a conserved protein interaction interface [95,96]. This study raises an interesting hypothesis regarding the common ancestral proteins for enzymes and pseudoenzymes. Perhaps the loss of catalytic function is tolerated given that a function of the ancestral protein is to facilitate protein binding in addition to catalyzing the enzymatic reaction [3,97]. When the catalytic function of the ancestral protein is lost through evolution, the pseudoenzymes retain their binding interface to primarily act as protein interaction domains [97]. p190RhoGAPs harbor three pseudoGTPase domains : the N terminal pseudoGTPase domain binds GTP without hydrolyzing while the other two pseudoGTPase domains do not bind nucleotides [98,99]. The functional importance of this conserved and high number of pseudoenzyme domains within one polypeptide is unknown.

## Pseudoenzymes and moonlighting proteins

The previous examples of pseudoenzymes have functions related to that of their catalytic counterparts, either by binding the same ligands or regulating their counterparts. However, over 300 proteins across the domains of life display moonlighting activity characterized by multiple distinct functions carried out by the same protein [100]. Crystallins, structural proteins found in eye lenses, are examples of moonlighting proteins [101]. In addition to their structural role, crystallins have various enzymatic functions: delta-crystallin as argininosuccinate lyase; epsilon-crystallin as lactate dehydrogenase; zeta-crystallin as quinone oxidoreductase; and eta-crystallin as aldehyde dehydrogenase [102–106]. The number of known moonlighting enzymes is growing, and the use of bioinformatic analysis holds promise as a screening method to identify potential moonlighting activity [107]. Combining this analysis with the computational modelling of protein interactions can guide experimental research. The evolution of pseudoenzymes also suggests an intriguing possibility for the identification of moonlighting activity in enzymes, as it is possible that enzyme homologues of pseudoenzymes may share the same function.

Pseudoenzymes arising from a catalytically active enzyme retain the overall structural fold to function. As proposed by Pils et al., the loss of catalytic amino acids in a pseudoenzyme may reduce selective pressure to conserve catalysis, and thus support alternative conformations and uses for the active site [3,95]. This may be the case of pseudoenzymes that play a role in binding the same ligand or modify the catalytic site for other functions. In extreme cases, like that of SelO, the catalytic site is repurposed for alternative catalytic activity. But fewer constraints on active site conformation may prove beneficial for other pseudoenzyme activities, including the formation of protein complexes [97,108].

The *Trypanosoma brucei* protein arginine methyltransferase pseudoenzyme (TbPMRT1 PRO) forms a complex with its enzymatic counterpart, TbPRMT1 ENZ. Like Fam20A, TbPMRT1 PRO forms a complex with TbPRMT1 ENZ to increase its partner's catalytic activity [109]. TbPMRT1 PRO harbors a variation in the Rossman fold that abolishes cofactor binding, hence it is a predicted pseudoenzyme with no catalytic activity [110]. Size exclusion chromatography coupled with multi-angle light scattering (SEC-MALS) and small-angle X-ray scattering (SEC-SAXS) suggest that the enzyme and pseudoenzyme form a heterotetramer [110]. Structural analysis reveals the presence of hydrophobic patches on TbPRMT ENZ that are buried upon oligomerization with TbPRMT PRO. Mutations of amino acid residues at the interface of TbPRMT ENZ and PRO disrupt oligomerization and methyltransferase activity. The altered Rossman fold in TbPRMT PRO, lacking a critical helix, tilts the adjacent α-helices, which facilitates dimerization with TbPRMT ENZ. The same structural change that aids in the oligomerization of TbPMRT1 PRO negates the catalytic activity. These studies demonstrate an interesting loss of enzymatic activity that occurred simultaneously with mutations promoting complex formation to improve the enzymatic activity of TbPRMT ENZ.

## Conclusion

The examples above demonstrate the utility of bioinformatic analysis and structural genomics in guiding experimental validation of pseudoenzyme function [111]. Sequence-based predictions alone may ignore the role of three-dimensional structure in protein activity [5,112]. Fortunately, the advent of powerful structural analysis tools such as AlphaFold has allowed more recent surveys to take three-dimensional structure into account. A study of the secreted effector proteins from fungal phytopathogen, *M. oryzae*, used TrRosetta structural algorithms to explore, among other things, the evolution of pseudoADP-ribosyl transferases by comparing their predicted structures to active counterparts. Notably, this survey also revealed several proteins with analogous structures but divergent sequences, suggesting that unrelated sequences might result in similar functions [95].

We are at an exciting time in history with new and exciting biology on the horizon of pseudoenzymes. Although we understand the functional role of some pseudoenzymes, we lack an understanding of the biochemical mechanisms of action. Experimental validation is limited by the identification of optimal conditions and physiological substrates, necessity for cofactors, and the understanding of catalytic mechanism/biochemical reaction. Weak or vestigial activity of pseudoenzymes *in vitro* may be artificial and may not reflect its function in cell. Some suggested methods to aid in the study of pseudoenzymes include immunoprecipitation and pulldown assays to identify interaction partners, intact mass analysis to detect post-translational modification, and structural and mutational analysis paired with biochemical assays to understand the molecular basis of activity. Conformational trapping of pseudoenzymes using monobodies or nanobodies is particularly useful to stabilize a single conformation for structural studies. Caution must be exercised when assaying immunoprecipitated pseudoenzymes, as contaminating enzymes may skew the analysis. A technique that is amenable to high-throughput screening is the SYPRO Orange thermal shift assay. Ligand binding increases the thermal stability of some proteins as seen with ATP binding to pseudokinases. Thermal stability of a protein can be quantified as a measure of increasing fluorescence upon protein denaturation and SYPRO orange binding to hydrophobic patches on proteins [113]. The study of the inverted orientation of ATP in the active site of the pseudokinase SelO illustrates the use of structure-guided hypothesis for potential alternative activities. Screening proteins for activity via predicted three-dimensional structures may provide guidance when investigating a potential pseudoenzyme, offering a compromise between testing for a selected number of activities rather than an impractically large number of potentials. The recent explosion in structural information from X-ray crystallography, cryo-electron microscopy, and AI-guided structure predictions greatly aids these studies. Unsurprisingly, the more in depth we study pseudoenzymes, the more capable we are of making an educated guess about the functional importance and molecular basis for the activity of understudied pseudoenzymes.

The pseudokinases are the most extensively studied pseudoenzyme family and a major target for drug discovery [15,114]. The modes of action of pseudokinases have parallels in other pseudoenzyme families (Table 1). Even those families that contain few or no pseudoenzymes are informative by suggesting constraints on pseudoenzyme evolution, demonstrating when loss of catalytic activity cannot be tolerated. As noted in Murphy et al. [1], the HECT E3 ubiquitin ligase family appears to have no pseudoenzyme counterparts, suggesting that the catalytic activity of these enzymes is essential to their cellular function. Further study of pseudoenzyme families is necessary to broaden our understanding of the multiple modes of action and cross-talk between families that contribute to the functional diversity and versatility of pseudoenzymes.

## Open Access

Open access for this article was enabled by the participation of the University of Texas Southwestern Medical Center in an all-inclusive *Read & Publish* agreement with Portland Press and the Biochemical Society under a transformative agreement with Individual.

## Abbreviations

ART: ADP-ribosyltransferase
CC1: Cys-based Class 1
DUSPs: dual-specificity phosphatases
ERK: extracellular regulated kinase
HUSH: human silencing hub
IFNλR1: interferon λ receptor 1
IRAK: interleukin-1 receptor-associated kinases
JAK: Janus kinase
MLKL: mixed lineage kinase domain-like
MPP8: M-phase phosphoprotein 8
OSR1: oxidative stress response kinase-1
POMK: protein-O mannose kinase
RIPK3: receptor-interacting serine–threonine kinase
SAXs: small angle X-ray scattering
SPAK: STE20/SPS1-related proline/alanine-rich kinase
STYX: serine/threonine/tyrosine interacting protein
TASOR: transgene activation suppressor protein
TKL: tyrosine kinase-like
YFP: yellow fluorescent protein
